# Predictors of COVID-19 in an outpatient fever clinic

**DOI:** 10.1371/journal.pone.0254990

**Published:** 2021-07-21

**Authors:** Frank Trübner, Lisa Steigert, Fabian Echterdiek, Norma Jung, Kirsten Schmidt-Hellerau, Wolfram G. Zoller, Julia-Stefanie Frick, You-Shan Feng, Gregor Paul

**Affiliations:** 1 Department of Gastroenterology, Hepatology, Pneumology and Infectious diseases, Klinikum Stuttgart, Stuttgart, Germany; 2 Department of Nephrology, Klinikum Stuttgart, Stuttgart, Germany; 3 Division of Infectious Diseases, Department I of Internal Medicine, University of Cologne, Cologne, Germany; 4 Institute of Medical Microbiology and Hygiene, University of Tübingen, Tübingen, Germany; 5 Institute for Clinical Epidemiology and Applied Biometry, University of Tübingen, Tübingen, Germany; 6 Department of Hospital Hygiene, Klinikum Stuttgart, Stuttgart, Germany; Mayo Clinic Minnesota, UNITED STATES

## Abstract

**Background:**

The objective of this study was to identify clinical risk factors for COVID-19 in a German outpatient fever clinic that allow distinction of SARS-CoV-2 infected patients from other patients with flu-like symptoms.

**Methods:**

This is a retrospective, single-centre cohort study. Patients were included visiting the fever clinic from 4^th^ of April 2020 to 15^th^ of May 2020. Symptoms, comorbidities, and socio-demographic factors were recorded in a standardized fashion. Multivariate logistic regression was used to identify risk factors of COVID-19, on the bases of those a model discrimination was assessed using area under the receiver operation curves (AUROC).

**Results:**

The final analysis included 930 patients, of which 74 (8%) had COVID-19. Anosmia (OR 10.71; CI 6.07–18.9) and ageusia (OR 9.3; CI 5.36–16.12) were strongly associated with COVID-19. High-risk exposure (OR 12.20; CI 6.80–21.90), especially in the same household (OR 4.14; CI 1.28–13.33), was also correlated; the more household members, especially with flu-like symptoms, the higher the risk of COVID-19. Working in an essential workplace was also associated with COVID-19 (OR 2.35; CI 1.40–3.96), whereas smoking was inversely correlated (OR 0.19; CI 0.08–0.44). A model that considered risk factors like anosmia, ageusia, concomitant of symptomatic household members and smoking well discriminated COVID-19 patients from other patients with flu-like symptoms (AUROC 0.84).

**Conclusions:**

We report a set of four readily available clinical parameters that allow the identification of high-risk individuals of COVID-19. Our study will not replace molecular testing but will help guide containment efforts while waiting for test results.

## Background

SARS-CoV-2 (severe acute respiratory syndrome coronavirus 2), the virus that causes coronavirus disease 2019 (COVID-19), rapidly spread all over the globe. The diagnosis primarily relies on molecular techniques, such as reverse transcriptase-polymerase chain reaction (RT-PCR) or antigen detection from nasopharyngeal swabs. Especially RT-PCR test results, considered as the gold standard, are often delayed due to transport, slow turnaround times and the requirement of a centralized laboratory [1]. But the decision to quarantine or to transfer the patient to an isolation ward must be taken immediately by physicians. Rapid identification of potential cases is essential for the containment of the virus [2]. However, a clinical differentiation between COVID-19 and diseases accompanied by flu-like symptoms is difficult to decide due to the often unspecific clinical presentation [3].

Several studies on prediction models have been performed, but most of them focused on predictors for patients in a hospital setting [4–6]. However, the majority of COVID-19 patients displays mild to moderate symptoms and hospitalization is often not required [7]. These patients more likely consult their general practitioner who would refer them to the local fever clinic.

Here we performed a study in the local fever clinic in Stuttgart, Germany, focusing on mild to moderate outpatient cases of COVID-19. Our study aimed to identify parameters that are readily available in an outpatient setting and help to identify patients with a high risk of SARS-CoV-2 infection.

## Materials and methods

### Study population

Stuttgart is the capital and largest city of the German state of Baden-Wuerttemberg with a population of around 635,000 people. Unlike other fever clinics which are associated with local hospitals, this fever clinic of Stuttgart was not part of a hospital but rather established by the local health authorities.

This single-center retrospective cohort study included all patients visiting this fever clinic from 4^th^ of April 2020 to 15^th^ of May 2020. Patients aged 18 or older were either self-referred or sent by their general practitioner in case they had common respiratory symptoms compatible with COVID-19 and/or a confirmed contact to a COVID-19 case.

### Data collection

Data on the type of symptoms, symptom onset, demographics, medical comorbidities, medication, predisposing risk factors, exposure and self-rated health were recorded via a standardized questionnaire used by the treating physician. The questionnaire was introduced in the fever clinic independently of this study to harmonize the workflow. It is shown in S1 Fig (English Version) and S2 Fig (German Version). In case of repeated visits of a patient, only data from the first visit was used for the analysis.

Self-rated health was recorded on a scale from one (excellent) to five (poor). Missing data was stated as such. The missing data analysis is shown in S1 Table. Missing data in the regression was dropped case-by-case.

#### Definition of risk factors and vital signs

Smoking was recorded in pack years (PY). If PY were missing, the number of cigarettes per day was documented. We created two categories for mild to moderate (≤5cig./day or ≤15PY) and heavy (>5cig./day or >15PY) smoking.

Essential workers (EW) were defined as people working in critical infrastructure such as retail, health care or police. The definition of contact persons followed the definitions by the Robert Koch Institute responsible for disease control and prevention. High-risk exposure is defined as contact to a COVID-19 case for more than 15 minutes and within distances of 1.5 meters or less without personal protective equipment (PPE). Low-risk exposure was defined as contact in a closed environment with a COVID-19 case for less than 15 minutes or at a distance more than 1.5 meters also without PPE. Exposure as a health care worker (HCW) providing care to a COVID-19 case wearing recommended PPE was looked at independently.

#### SARS-CoV-2 testing and reporting to public health department

SARS-CoV-2 testing was performed on nasopharyngeal swabs via RT-PCR on the Cobas 6800 system (Cobas, Roche, Basel, Switzerland). As obliged by the German Infection Protection Act (Infektionsschutzgesetz), patients with suspected or confirmed COVID-19 were reported to the local health department.

#### Ethics

The study was approved by the ethics committee of the Landesärztekammer Baden-Württemberg, Stuttgart, Germany (vote F-2020-067). Because of the retrospective and anonymized approach, the need for informed consent was waived by the local ethics committee.

#### Statistical analysis

Continuous data was expressed as mean, median and interquartile range (IQR) while categorical variables were reported as number (n) and percentage (%). Statistical differences across positive/negative SARS-CoV-2 patients were determined using Student’s t-test for continuous variables. Chi-squared test or Fisher’s exact test were performed for categorical variables. Chi-Squared test was used when all cells had at least ten observations. Otherwise, the Fisher’s exact test was applied. Wilcoxon rank-sum test was used to compare BMI between groups. SARS-CoV-2 test result (positive / negative) was used as the dependent variable in logistic regression. Candidate risk factors were entered first individually to assess their relationship with positive SARS-CoV-2 tests. Multivariate modelling was informed both by prior knowledge of the risk factors as well as the results of bivariate models. Logistic regression results are reported as odds ratios (OR) with 95% confidence intervals (95% CI). Reported p-values are 2-tailed, with P≤0.05 being considered statistically significant. Lastly, area under the receiver operating characteristics (AUROC) was used to examine the value of the models discriminating between patients with positive and negative SARS-CoV-2 tests.

The flow diagram was created using LibreOffice Draw (Version 6.4.4, The Document Foundation, Berlin, Germany). Visualization of data was performed using GraphPad Prism (Version 7.0.0, GraphPad Software, San Diego, California USA). Regressions were modelled using STATA 13 (StataCorp. 2013, Stata Statistical Software: Release 13, College Station, TX: StataCorp LP).

## Results

### Study population and clinical characteristics

**Fig 1:**
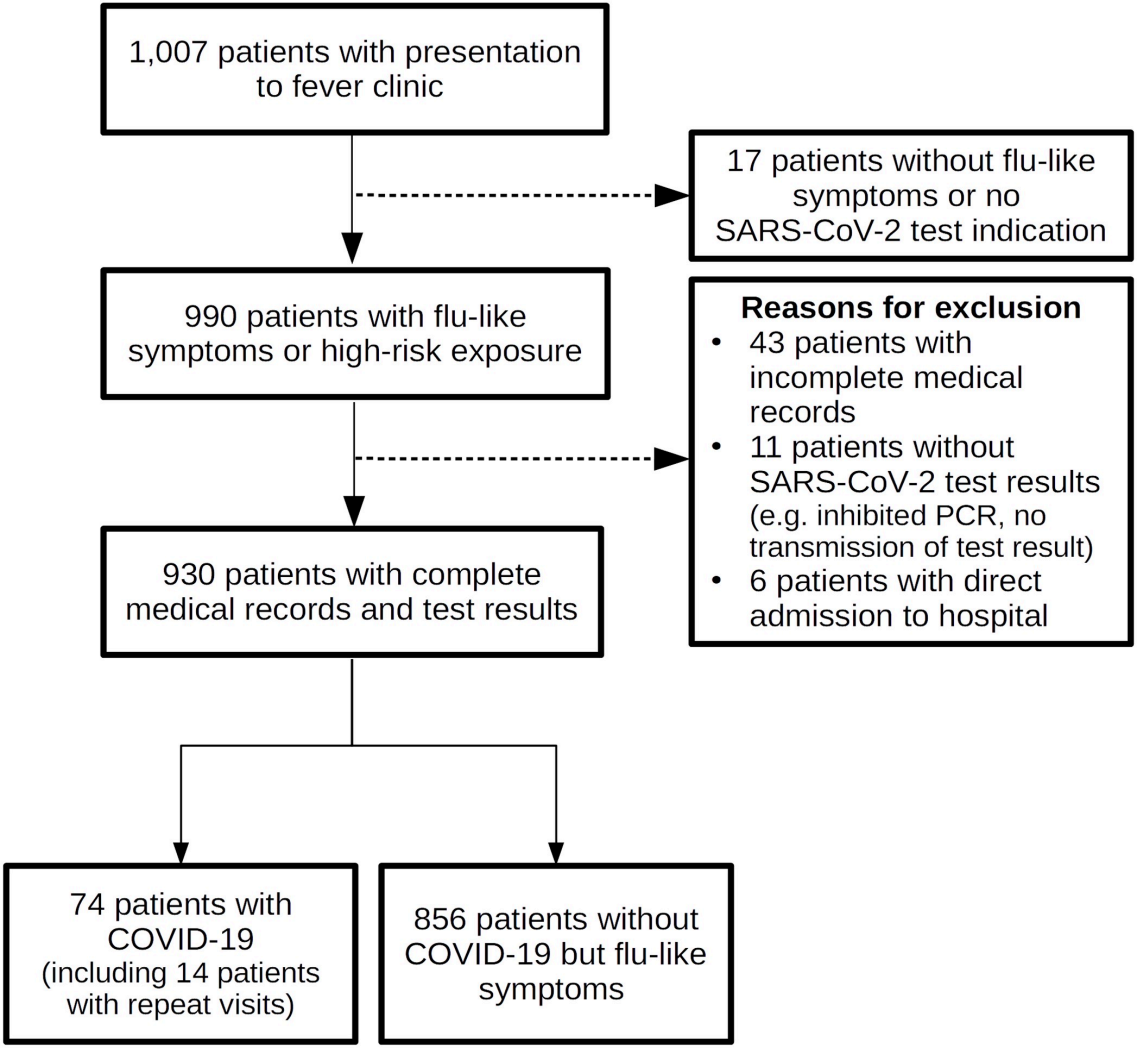
Flow diagram showing study population selection. All patients visiting a fever clinic from 4th of April 2020 to 15th of May 2020 with flu-like symptoms or high-risk exposure were included in this study. In case of repeated visits only data from the first visit was used.

A flow diagram showing the study population selection is shown in Fig 1. In total 930 patients were included in the final analysis. Among them, 74 patients (8%) revealed a positive RT-PCR for SARS-CoV-2.

Patient characteristics and demographics are summarized in Table 1. Median age was 44 years (IQR 34–55) for COVID-19 patients and 41 years (IQR 30–54) for those with a negative SARS-CoV-2 test result. There was no difference between groups regarding age or gender. The median BMI of patients with COVID-19 was significantly higher than in the reference group (27 vs. 25.5 kg/m^2^; p-value = 0.0165). At least one fifth of the patients in both groups had one comorbidity or more. However, no difference was seen for comorbidities or medication intake.

**Table 1:** Demographics and comorbidities for patients with and without COVID-19.

	SARS-CoV-2 positive N = 74	SARS-CoV-2 negative N = 856	p-value
	N / N_total_ (%)	N / N_total_ (%)	
**Demographics**			
Age, median (IQR), years	44 (34–55)	41 (30–54)	0.20
Male sex	30 / 74 (40.5)	350 / 855 (40.9)	0.95
BMI, median (IQR)	27.0 (23.5–32.5)	25.50 (22–29)	**0.02**
Pregnancy	1 / 41 (2.44)	3 / 503 (0.6)	0.42
**Comorbidities**			
At least one comorbidity	16 / 73 (21.9)	278 / 856 (32.5)	0.06
Asthma	5 / 73 (6.9)	100 / 856 (11.7)	0.25
COPD	0 / 73 (0)	17 / 856 (2)	0.39
Arterial hypertension	10 / 73 (13.7)	138 / 856 (16.1)	0.59
Diabetes	4 / 73 (5.5)	50 / 856 (5.8)	1
Chronic kidney disease	0 / 73 (0)	7 / 856 (0.8)	1
Active cancer	0 / 73 (0)	10 / 856 (1.2)	1
Coronary artery disease	0 / 73 (0)	16 / 856 (1.9)	0.63
Liver cirrhosis	0 / 73 (0)	4 / 856 (0.5)	1
HIV	1 / 73 (1.4)	5 / 856 (0.6)	0.39
Connective tissue disease	0 / 73 (0)	10 / 856 (1.2)	1
**Medication**			
ACE-Inhibitors	4 / 70 (5.7)	40 / 852 (4.7)	0.72
AT-II receptor blockers	3 / 70 (4.3)	47 / 852 (5.5)	0.68

IQR; interquartile range, CI; confidence interval, BMI; Body Mass Index, COPD; chronic obstructive pulmonary disease, HIV; human immunodeficiency virus, ACE; angiotensin converting enzyme, AT-II; angiotensin II

### Flu-like symptoms and vital signs as predictors for COVID-19

**Fig 2:**
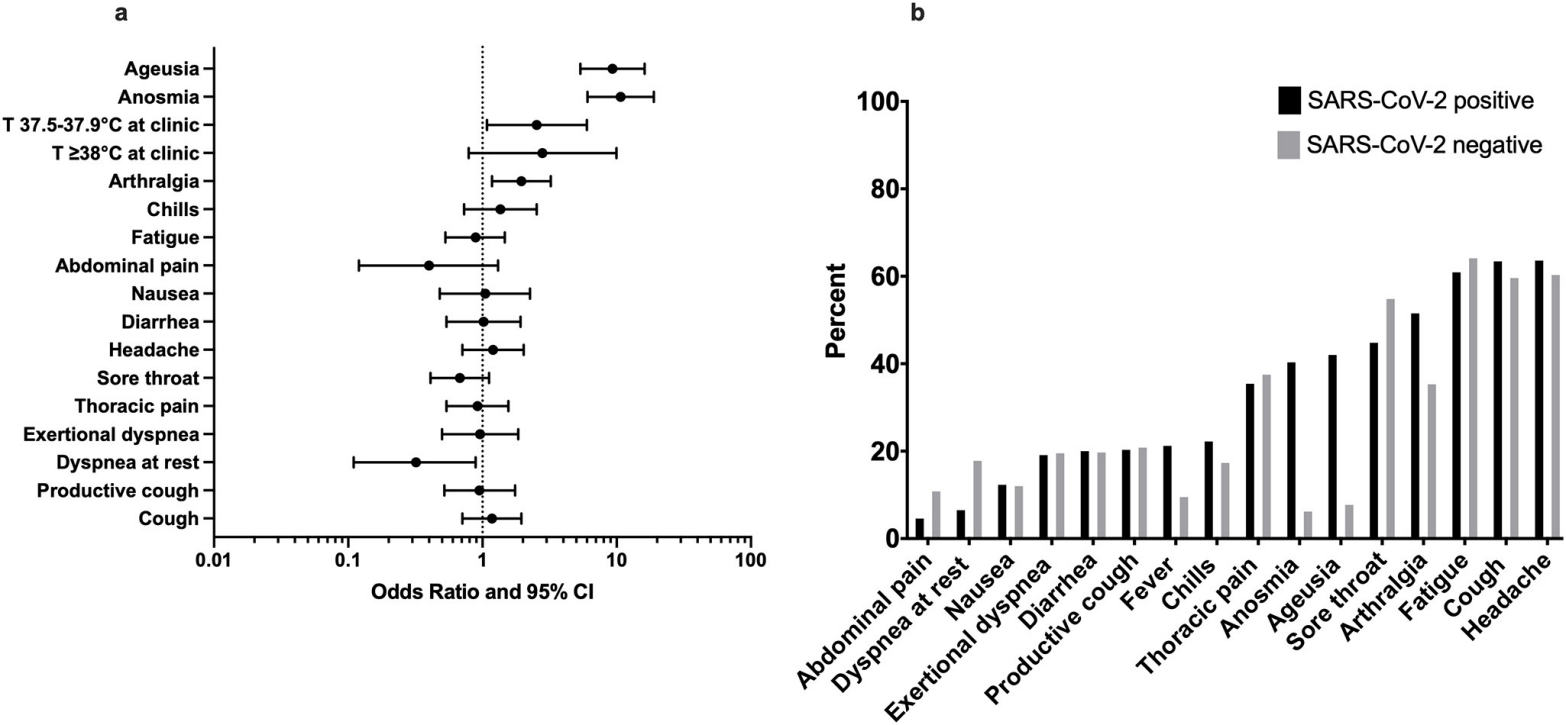
Symptoms as predictors of COVID-19. (A) Fig 2a shows the forest plot depicting the association between symptoms and the risk for COVID-19. The reference group for all symptoms is the group, in which the respective symptom was not present. Fisher’s exact test was used for the calculation. X-axis shows relative increase in risk of COVID-19 as odds ratio. Y-axis denotes individual symptoms. Results are expressed with 95% confidence intervals. (B) Fig 2b shows the relative frequency of individual symptoms in patients with COVID-19 and with other flu-like symptoms. X-axis shows individual symptoms while the Y-axis shows relative frequency in percentage. Abbreviations: T; Temperature.

While most symptoms did not allow any discrimination between SARS-CoV-2 positive and negative patients, some were identified as predictors for COVID-19 after adjustment for age and gender (Fig 2A). These were anosmia (OR 10.71; CI 6.07–18.9; p-value <0.001), ageusia (OR 9.3; CI 5.36–16.12; p-value <0.001) and arthralgia (OR 1.95; CI 1.18–3.23; p-value 0.01). Dyspnea at rest was less common in COVID-19 patients (OR 0.32; CI 0.11–0.89; p-value 0.03). The most common symptoms reported by COVID-19 patients were headache (64%), fatigue (60%), fever (53%), arthralgia (51%) and sore throat (45%), followed by ageusia (42%) and anosmia (40%) (Fig 2B). Detailed data is shown in S2 Table.

Patients with COVID-19 had more often subfebrile temperatures (37.5–37.9°C) than patients without COVID-19. This was valid for the measured temperature at home (OR 2.36; CI 1.16–4.79; p-value 0.02) as well as the temperature measurement at the fever clinic (OR 2.54; CI 1.08–5.99; p-value 0.03). No difference between groups was found for oxygen saturation (median 97% vs 98%, p-value 0.41), systolic (median 120mmHg vs 125mmHg, p-value 0.13) or diastolic blood pressure (median 80mmHg vs 80mmHg, p-value 0.16) nor heart rate (median 85.5 vs 83, p-value 0.22).

### Social risk factors for COVID-19

The results are shown in Table 2. High-risk exposure to an individual with COVID-19 was found to be a strong risk factor for COVID-19 (OR 12.20; CI 6.80–21.90; p-value <0.001). Exposure to a case living in the same household was also a predictor for COVID-19 acquisition (OR 4.14; CI 1.28–13.33; p-value = 0.02), which was not the case when the exposure occurred knowingly at a (medical or non-medical) workplace with or without PPE. Still, essential workers were at higher risk (OR 2.35; CI 1.40–3.96; p-value = 0.001). Living in a two-person household showed a significantly higher risk compared to a one-person household (OR 2.82; CI 1.06–7.53; p-value = 0.04). The risk was even higher for a household with more than two people (OR = 4.72; CI 1.82–12.26; p-value = 0.001). This difference was more pronounced when household members also reported flu-like symptoms. Active smoking was found significantly less common in patients with COVID-19 (OR 0.19; CI 0.08–0.44; p-value <0.001). This was true for heavy smokers (more than 15PY or more than 5 cigarettes a day) as well as mild to moderate smokers.

**Table 2:** Social risk factors for COVID-19 in an outpatient fever clinic.

	SARS-CoV-2 positive		SARS-CoV-2 negative					
	N = 74		N = 856					
	N / N_total_	(%)	N / N_total_	(%)	Odds ratio	95% CI	p-value
**Essential workers**	35 / 67	52.2	279 / 835	33.4	2.36	1.40–3.96	**0.001**
**Type of exposure**							
No contact	22 / 66	33.3	671 / 853	78.7	Reference group		
High risk	40 / 66	60.6	123 / 853	14.4	12.20	6.79–21.90	**< 0.001**
Low risk	3 / 66	4.6	40 / 853	4.7	2.48	0.71–8.73	0.16
HCW with PPE	1 / 66	1.5	19 / 853	2.2	1.88	0.24–14.80	0.55
**Contact person**							
Workplace	4 / 41	9.8	35 / 155	22.6	Reference group		
Medical environment	11 / 41	26.8	40 / 155	25.8	1.95	0.55–6.90	0.30
Same household	22 / 41	53.7	44 / 155	28.4	4.14	1.28–13.33	**0.02**
Other	4 / 41	9.8	36 / 155	23.2	0.67	0.15–3.04	0.60
**Household members**							
One-person household	5 / 68	7.4	187 / 844	22.2	Reference group		
2	24 / 68	35.3	328 / 844	38.9	2.82	1.06–7.53	**0.04**
> 2	39 / 68	57.4	329 / 844	39	4.72	1.82–12.26	**0.001**
**Symptomatic household members**							
None	27 / 66	40.9	623 / 841	74	Reference group		
One	28 / 66	42.4	189 / 841	22.5	3.48	2.00–6.07	**< 0.001**
> 1	11 / 66	16.7	29 / 841	3.5	8.65	3.90–19.17	**< 0.001**
**Smoking status**							
Non-smoker	61 / 67	91.0	559 / 849	65.8	Reference group		
Smoker	6 / 67	9.0	290 / 849	34.2	0.19	0.08–0.44	**< 0.001**
Heavy smoking	1 / 66	1.5	104 / 839	12.4	0.08	0.01–0.62	**0.015**
Moderate smoking	4 / 66	6.1	175 / 839	20.9	0.21	0.08–0.60	**0.003**
**Report**	27 / 60	45.0	125 / 856	13.65	6.33	3.65–10.97	**< 0.001**
**Adjusted for**							
+age, gender					6.92	3.94–12.16	**< 0.001**
+age, gender, history of exposure					5.22	2.81–9.72	**< 0.001**
+age, gender, symptoms					6.38	3.30–12.33	**< 0.001**
+age, gender, symptoms, history of exposure					4.39	2.12–9.11	**< 0.001**

The table shows the results for the logistic regression analysis for exposure and social background as risk factors for COVID-19 after adjustment for age and gender. Essential workers (e.g., retail, police, or HCW) were of systemic importance during the first lockdown in spring 2020. High risk exposure is defined as having had face-to-face contact with a COVID-19 case within 1.5 meters and more than 15 minutes. Low risk exposure is defined as having had contact with a COVD-19 case in a closed environment or within 1.5 meters for less than 15 minutes. Exposure as a HCW providing care to a COVID-19 case wearing recommended PPE was looked at separately. Examples for a contact person at a medical environment might be HCW with contact to infected colleagues at work or to patients with or without appropriate PPE. Mild to moderate smokers (≤5cig./day or ≤15PY) and heavy (>5cig./day or >15PY) smokers among active smokers were compared to non-smokers.

CI; confidence interval, HCW; health care worker, PPE; personal protective equipment

### Development of a risk model

**Fig 3:**
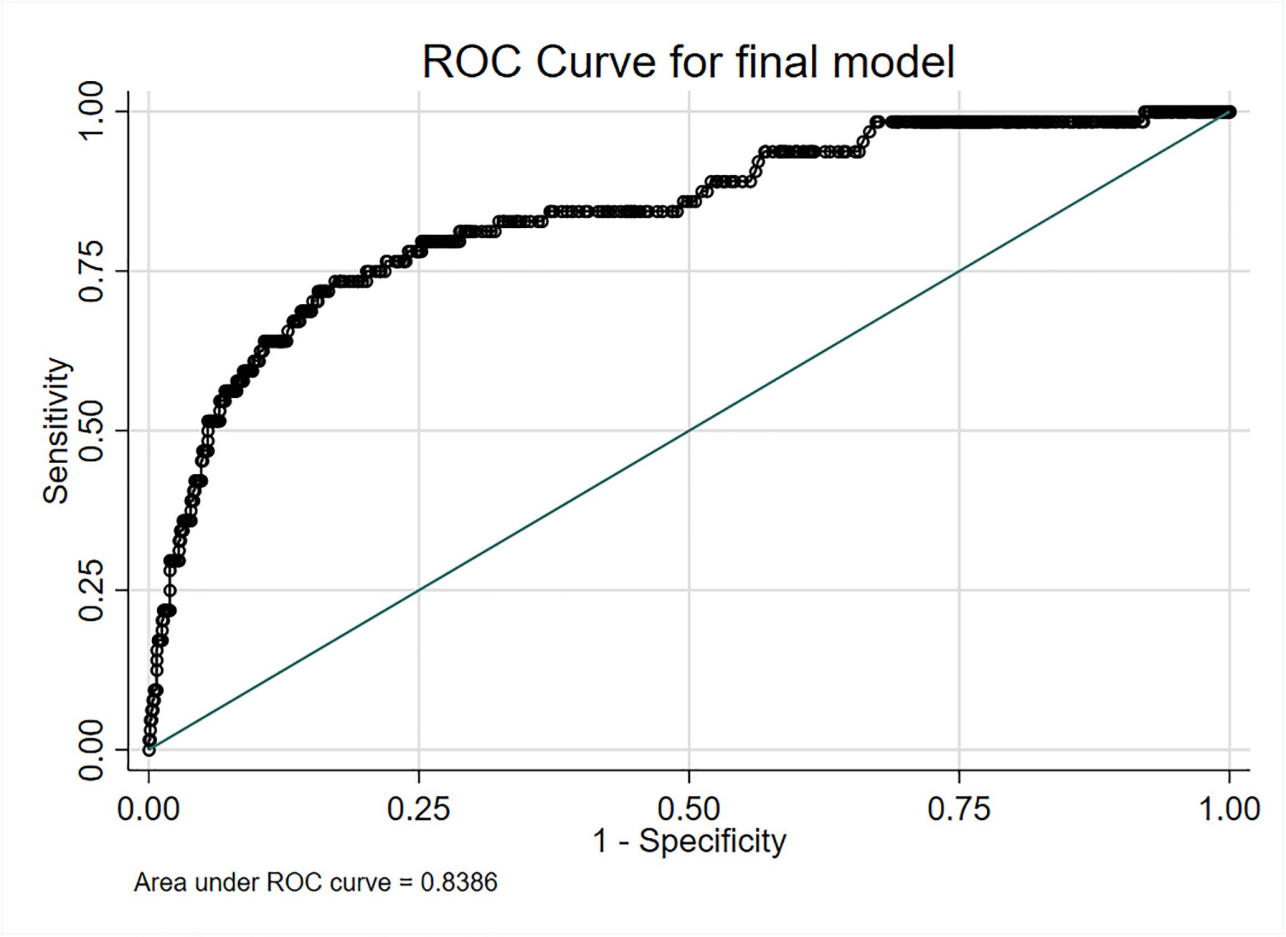
Multivariable prediction model for COVID-19. Performance of the multivariable prediction model for COVID-19 including the variables age, gender, anosmia, ageusia, symptomatic household members and smoking using an area under the receiver operating characteristic (AUROC) curve.

Anosmia and ageusia (alone or in combination) had suboptimal performance as sole predictors of COVID-19 (AUROC 0.67–0.74). The same is true for high-risk exposure (AUROC 0.74). The best multivariable model was achieved when age, gender, anosmia, ageusia, smoking status and contact to symptomatic household members were included as parameters. This model achieved an excellent performance with an AUROC of 0.84 (Fig 3). The model fit of the logistic regression is shown in S3 Table.

## Discussion

There is an expanding access to fast and reliable laboratory tests for the diagnosis of COVID-19. However, it is still a challenge in emergency departments or in other outpatient settings to decide which patient with flu-like symptoms requires instant isolation and contact tracing.

In our study, we identified several predictors for COVID-19 in outpatient setting. We also developed a model that solely depends on information that can be gathered without invasive procedures.

Sun et al. published prediction models based on cases at a tertiary hospital in Singapore [4]. Their models with good performance (area under curve [AUC] >0.8) were dependent on laboratory tests or radiographic scans though. A model developed solely on non-invasively gathered information turned out to be non-satisfactory (AUC 0.65). In our cohort, COVID-19 related symptoms that allowed discrimination from other flu-like diseases were anosmia and ageusia, which were not included in their model. Loss of taste and smell are distinct features of COVID-19 and have shown to be good predictors of the disease [6, 8]. We also identified by univariate analysis that patients with COVID-19 had more often arthralgia and subfebrile temperatures (37.5–37.9°C), but less often dyspnea at rest. Patients with COVID-19 were less likely to be smokers, which might explain why they were less likely to report dyspnea at rest. Though, those symptoms are quite unspecific and eventually did not add any predictive value to our model.

Interestingly, smokers are at a reduced risk of SARS-CoV-2 infection in our study, an observation that was also confirmed by other groups [9]. The reason for this is largely unknown. Mechanistically, the angiotensin converting enzyme (ACE)-2 receptor, the entry site for SARS-CoV-2, is upregulated in the respiratory epithelium of smokers [10]. But it is unclear whether the frequency of ACE2 receptors on mucosal cells has an influence on disease susceptibility [11]. Certainly, ACE inhibitors or angiotensin 2 receptor blockers, which change ACE2 expression, are not linked to SARS-CoV-2 susceptibility, as proven in our and several other studies [12, 13]. More studies are required to answer the question if cigarette smoking itself will lead to a reduced susceptibility to SARS-CoV-2 infection or if an underlying behavioral trait (e.g., leaving closed environments for smoking more often) will influence the risk. Although our study implies that smokers are less prone to SARS-CoV-2 infection, several other studies have shown that once a patient is infected, smoking is a predictor for severe disease and in-hospital mortality [14–16]. Therefore, we certainly do not suggest starting or continuing smoking during the ongoing pandemic.

BMI was also identified as a predictor for COVID-19, which falls in line with the published literature, linking obesity to respiratory infections as a general and to COVID-19 [17, 18]. Like smoking, the mechanisms for the heightened susceptibility are still elusive. Likewise, patients with obesity are at a high risk for mortality of COVID-19 [19].

Unsurprisingly, the more intense the exposure to COVID-19 cases, the higher the risk for infection. We show that the definition of contact persons of the European Centre for Disease Prevention and Control (ECDC), which was adopted by the German Robert-Koch-Institute, is helpful in determining the associated risk of infection [20]. High-risk exposures were strong predictors of COVID-19. As shown in other studies, the risk for SARS-CoV-2 infection is higher among household contacts than that in health care or other settings [21]. Having contact to a COVID-19 case as a HCW with appropriate PPE was not associated with a risk for SARS-CoV-2 infection in our study. This is mostly attributable to the small sample size of this subgroup and the protective effect of PPE. By contrast, working as an EW (including HCW) was significantly associated with infection risk which was also shown in other studies [22]. At the time of data collection, the face mask mandate in hospitals in Germany was not universally introduced. Therefore, many infections occurred between HCW. A higher occupational risk for SARS-CoV-2 acquisition among HCW is well known [23]. This fact should be acknowledged in times of discussions of allocation of SARS-CoV-2 vaccines to the public.

We show that symptoms and history of exposure are the strongest predictors for COVID-19. Still, after adjusting for these factors, it was possible for the treating physician to identify individuals with COVID-19 more often as would have been possible by chance. Therefore, as good as a prediction model might theoretically be, it will never replace molecular testing and the practical knowledge of an experienced physician.

The strength of our study is the large cohort and the agreement of our data with published studies, proofing to be a reliable data set despite the retrospective approach. Our predictors and model depend solely on non-invasive parameters, allowing for quick identification of individuals at risk.

The retrospective approach is a limitation to our findings. The accuracy of the data depends on meticulous documentation in times with a high level of stress during the beginning of the pandemic. Reporting bias is another limiting factor since some patients may have exaggerated their symptoms to receive a test. Furthermore, in some subgroups there were only a limited number of events (e.g., smoking, type of exposure) and multiple subgroup analysis increases the likelihood of a statistically significant false positive result.

In summary we identified that anosmia, ageusia, history of exposure, as well as smoking and BMI are strong predictors of COVID-19 in an outpatient setting. Especially the strong inverse correlation of smoking with COVID-19 warrants further research. Identifying the underlying mechanism might help to find preventive mechanisms.

In times of scarce hospital beds and over-crowded emergency departments and family practices, the identified predictors of COVID-19 provide help in the decision process for whom isolation and contact tracing is warranted.

## Supporting information

S1 Fig Questionnaire in English. (TIF)

S2 Fig Questionnaire in German. (TIF)

S1 Table Missing data analysis. (DOCX)

S2 Table Symptoms as predictive risk factors for COVID-19. (DOCX)

S3 Table Predictive accuracy and model fit of logistic regression models adjusted for age and gender. (DOCX)
