# Supplementary figures and images for: Predictors of COVID-19 in an outpatient fever clinic
Source: PLoS One. 2021 Jul 21;16(7):e0254990. doi: 10.1371/journal.pone.0254990 (PMC8294531; doi:10.1371/journal.pone.0254990)

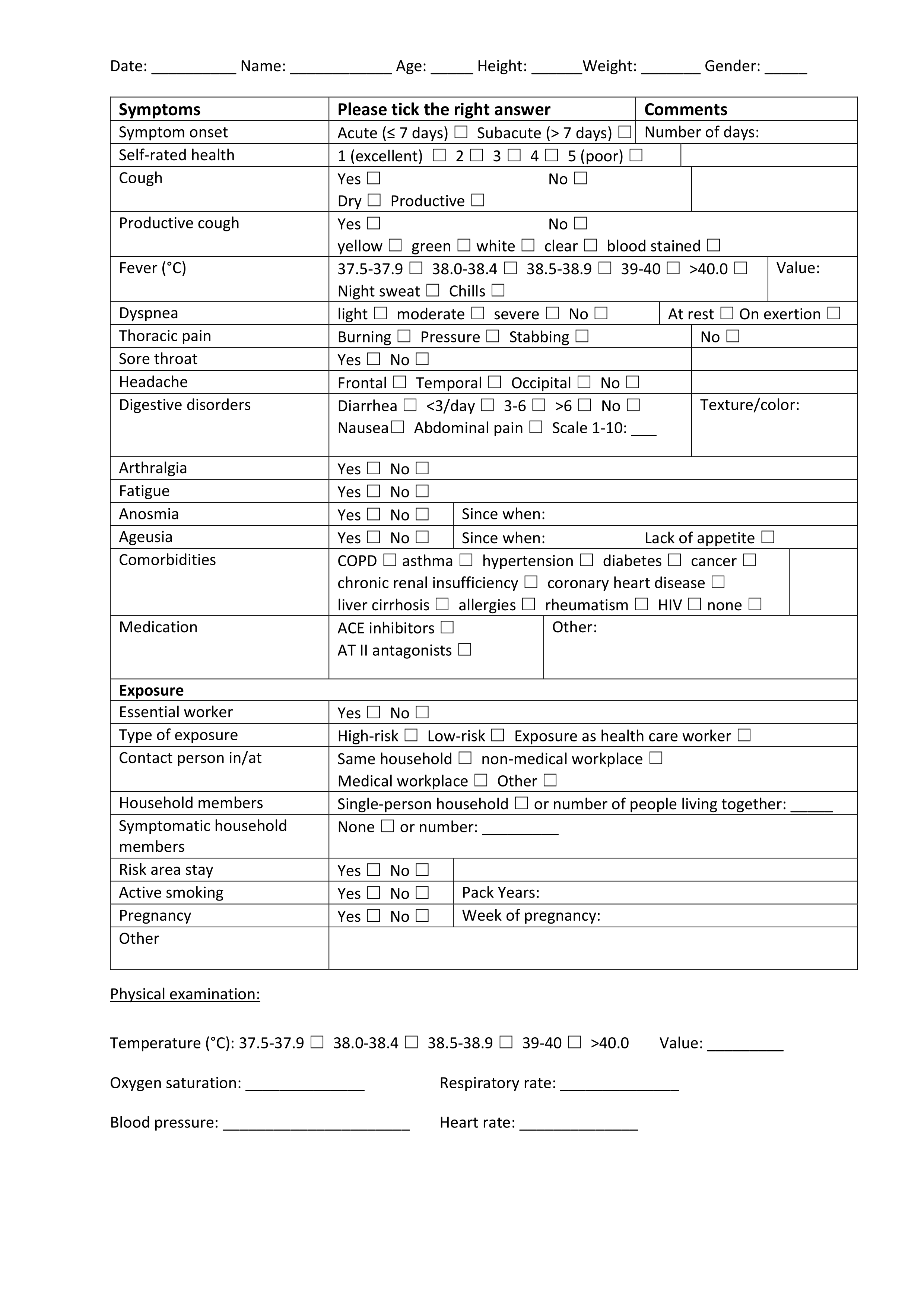

Supplement: S1 Fig — (TIF) [file pone.0254990.s001.tif]

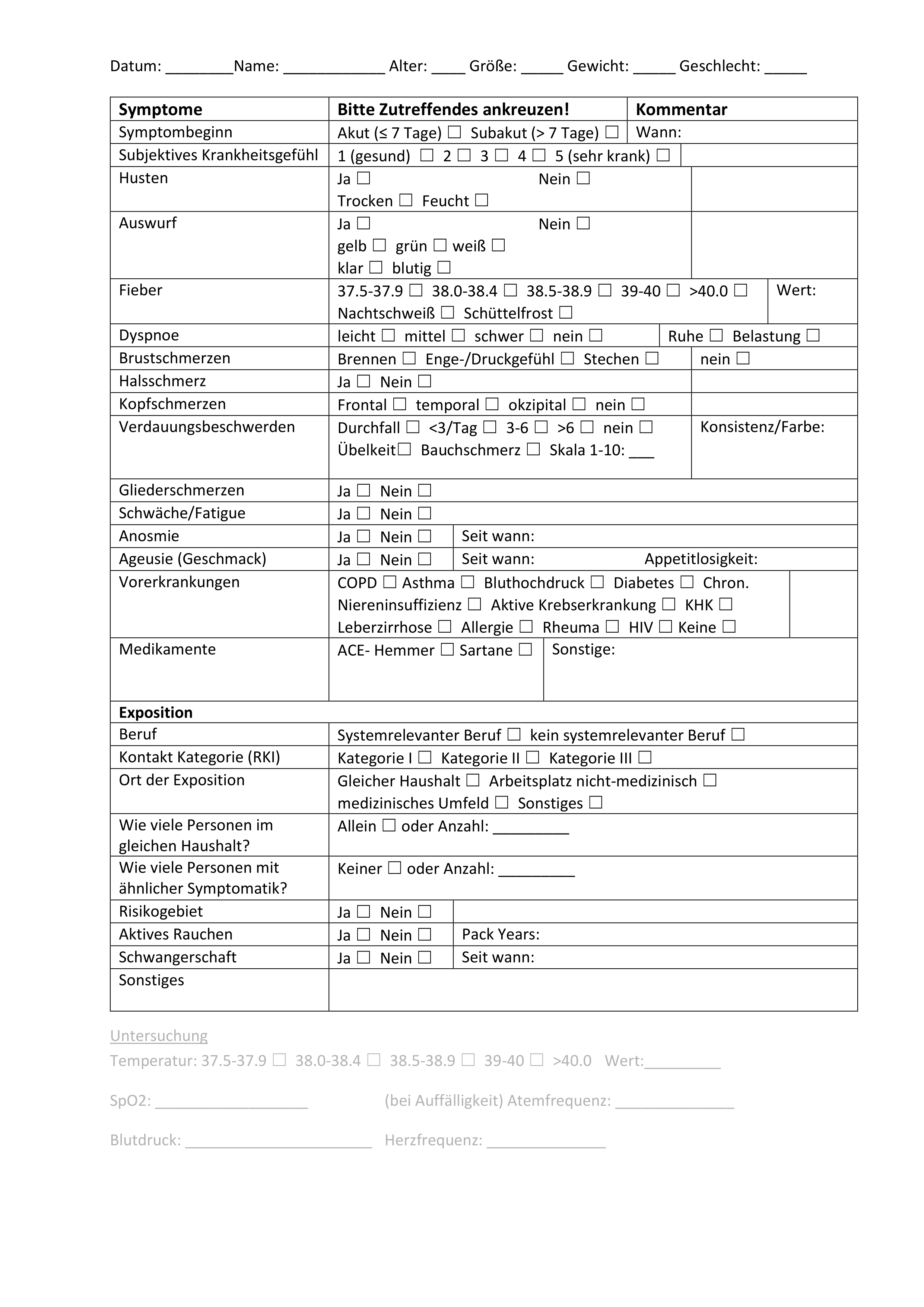

Supplement: S2 Fig — (TIF) [file pone.0254990.s002.tif]
