# Supplementary material for: Predictors of COVID-19 in an outpatient fever clinic
Source: PLoS One. 2021 Jul 21;16(7):e0254990. doi: 10.1371/journal.pone.0254990 (PMC8294531; doi:10.1371/journal.pone.0254990)
Supplement: S1 Table — (DOCX) [file pone.0254990.s003.docx]

| **S1 Table. Missing data analysis.** Causes: 1. Repeated consultation: In case of repeated visits of a patient to the fever clinic, only data from the first visit was used for the analysis.  2. Follow-up censoring: This censoring concerns the contact person category which was only documented if the patient reported any kind of contact/exposure and the clinical temperature which was only included in the analysis when no antipyretic intake was reported. 3. Incomplete documentation was due to illegible/no documentation, limitations concerning the physical examination (obesity, nail polish) and language barriers. | | | | |
| --- | --- | --- | --- | --- |
|  | **SARS-CoV-2 positive**  **N_total_ = 74** | | **SARS-CoV-2 negative**  **N_total_ = 856** | |
|  | **N_missing_** | **Cause** | **N_missing_** | **Cause** |
| Age | 0 | - | 1 | 3 |
| Gender | 0 | - | 3 | 3 |
| BMI | 13 | 1, 3 | 101 | 3 |
| Medication | 4 | 1, 3 | 4 | 3 |
| Comorbidities | 1 | 1 | 0 | - |
| Symptom onset | 5 | 1, 3 | 51 | 3 |
| Self-rated health | 16 | 1, 3 | 102 | 3 |
| Arthralgia | 8 | 1, 3 | 1 | 3 |
| Anosmia | 7 | 1, 3 | 3 | 3 |
| Ageusia | 5 | 1 | 2 | 3 |
| Cough | 3 | 1 | 0 | - |
| Productive cough | 5 | 1, 3 | 0 | - |
| Chills | 11 | 1, 3 | 4 | 3 |
| Dyspnoea at rest | 12 | 1, 3 | 36 | 3 |
| Exertional dyspnoea | 11 | 1, 3 | 37 | 3 |
| Thoracic pain | 9 | 1, 3 | 1 | 3 |
| Sore throat | 7 | 1, 3 | 0 | - |
| Headache | 8 | 1, 3 | 0 | - |
| Diarrhoea | 9 | 1, 3 | 0 | - |
| Nausea | 9 | 1, 3 | 0 | - |
| Abdominal pain | 9 | 1, 3 | 1 | 3 |
| Fatigue | 5 | 1 | 1 | 3 |
| Essential workers | 7 | 1, 3 | 21 | 3 |
| Type of exposure | 8 | 1, 3 | 3 | 3 |
| Contact person | 33 | 2 | 701 | 2 |
| Household members | 6 | 1, 3 | 12 | 3 |
| SHM | 8 | 1, 3 | 15 | 3 |
| Smoking status | 7 | 1, 3 | 7 | 3 |
| Mild-moderate, heavy smoker | 8 | 1 | 17 | 3 |
| SpO_2_ | 16 | 1, 3 | 20 | 3 |
| SBP | 16 | 1, 3 | 31 | 3 |
| DBP | 20 | 1, 3 | 57 | 3 |
| HR | 16 | 1, 3 | 15 | 3 |
| Temperature measured at home | 14 | 1, 3 | 3 | 3 |
| Clinical temperature | 27 | 1, 2, 3 | 100 | 2, 3 |
| Report | 14 | 1 | 0 | - |

BMI; Body Mass Index, SBP; Systolic blood pressure, DBP; Diastolic blood pressure, HR; heart rate, SHM, symptomatic household member
