# Supplementary material for: Predictors of COVID-19 in an outpatient fever clinic
Source: PLoS One. 2021 Jul 21;16(7):e0254990. doi: 10.1371/journal.pone.0254990 (PMC8294531; doi:10.1371/journal.pone.0254990)
Supplement: S2 Table — (DOCX) [file pone.0254990.s004.docx]

| **S2 Table. Symptoms as predictive risk factors for COVID-19.**  Logistic regression adjusted for age and gender. The reference group for all symptoms is the absence of this symptom. | | | | | | | | |
| --- | --- | --- | --- | --- | --- | --- | --- | --- |
|  | **SARS-CoV-2 positive**  **N = 74** | | **SARS-CoV-2 negative**  **N = 856** | |  |  |  | |
|  | **N / N_total_** | **(%)** | **N/N_total_** | **(%)** | **Odds ratio** | **95% CI** | **p-value*** | |
| **Symptom onset** | | | | | | | | |
| ≤ 7 days | 48 / 69 | 69.6 | 562 / 805 | 69.8 | Reference group | | | |
| > 7 days | 21 / 69 | 30.4 | 243 / 805 | 30.2 | 0.99 | 0.58 - 1.70 | | 0.98 |
| **Self-rated health** |  |  |  |  |  |  | |  |
| 1 | 8 / 58 | 13.8 | 141 / 754 | 18.7 | Reference group | | | |
| 2 - 5 | 50 / 58 | 86.2 | 613 / 754 | 81.3 | 1.48 | 0.69 - 3.21 | | 0.32 |
| **Arthralgia** | 34 / 66 | 51.5 | 302 / 855 | 35.3 | 1.95 | 1.18 - 3.23 | | **0.01** |
| **Anosmia** | 27 / 67 | 40.3 | 53 / 853 | 6.2 | 10.71 | 6.07 - 18.90 | | **< 0.001** |
| **Ageusia** | 29 / 69 | 42.0 | 66 / 854 | 7.7 | 9.30 | 5.36 - 16.12 | | **< 0.001** |
| **Cough** | 45 / 71 | 63.4 | 510 / 856 | 59.6 | 1.18 | 0.71 - 1.95 | | 0.52 |
| **Productive cough** | 14 / 69 | 20.3 | 178 / 856 | 20.8 | 0.95 | 0.52 - 1.75 | | 0.88 |
| **Chills** | 14 / 63 | 22.2 | 147 / 852 | 17.3 | 1.36 | 0.73 - 2.54 | | 0.33 |
| **Dyspnoea at rest** | 4 / 62 | 6.5 | 146 / 820 | 17.8 | 0.32 | 0.11 - 0.89 | | **0.03** |
| **Exertional dyspnoea** | 12 / 63 | 19.1 | 160 / 819 | 19.5 | 0.96 | 0.50 - 1.85 | | 0.91 |
| **Thoracic pain** | 23 / 65 | 35.4 | 321 / 855 | 37.5 | 0.92 | 0.54 - 1.56 | | 0.75 |
| **Sore throat** | 30 / 67 | 44.8 | 469 / 856 | 54.8 | 0.68 | 0.41 - 1.12 | | 0.13 |
| **Headache** | 42 / 66 | 63.6 | 516 / 856 | 60.3 | 1.20 | 0.71 - 2.03 | | 0.50 |
| **Diarrhea** | 13 / 65 | 20.0 | 169 / 856 | 19.7 | 1.02 | 0.54 - 1.92 | | 0.95 |
| **Nausea** | 8 / 65 | 12.3 | 103 / 856 | 12.0 | 1.05 | 0.48 - 2.26 | | 0.91 |
| **Abdominal pain** | 3 / 65 | 4.6 | 92 / 855 | 10.8 | 0.40 | 0.12 - 1.31 | | 0.13 |
| **Fatigue** | 42 / 69 | 60.9 | 548 / 855 | 64.1 | 0.89 | 0.53 - 1.47 | | 0.64 |

* Bold indicates a p-value ≤ 0.05.
