# Supplementary material for: Predictors of COVID-19 in an outpatient fever clinic
Source: PLoS One. 2021 Jul 21;16(7):e0254990. doi: 10.1371/journal.pone.0254990 (PMC8294531; doi:10.1371/journal.pone.0254990)
Supplement: S3 Table — (DOCX) [file pone.0254990.s005.docx]

| **S3 Table. Predictive accuracy and model fit of logistic regression models adjusted for age and gender.** SARS-CoV-2-PCR test positivity is the dependent variable. Measured temperature at home included the categories 37.5-37.9°C and ≥38.0°C; exposure grade included no known exposure, low-risk, and high-risk exposure; symptomatic household members included no household member, one household member and more than one household member. | | | | |
| --- | --- | --- | --- | --- |
| **Predictors** | **N_total_  in model** | **AUROC value** | **Hosmer-Lemeshow Chi-squared** | **LM Chi-squared** |
| Anosmia | 923 | 0.6618 | - | 53.65 |
| Ageusia | 925 | 0.6791 | - | 54.5 |
| Measured temperature at home | 913 | 0.5858 | - | 6.47 |
| Smoking status | 905 | 0.6306 | - | 24.16 |
| Anosmia + ageusia | 923 | 0.7018 | 0.9923 | 67.07 |
| Anomia + ageusia + smoking | 902 | 0.7654 | 0.8946 | 83.94 |
| **Model 1:**  **Anosmia + ageusia + smoking + home measured temperature** | 888 | 0.8145 | 0.3578 | 88.7 |
| Exposure grade | 919 | 0.7434 | - | 68.33 |
| Symptomatic household members | 907 | 0.6773 | - | 34.39 |
| **Model 2:** |  |  |  |  |
| **Exposure grade + symptomatic**  **household members** | 904 | 0.771 | 0.6844 | 76.83 |
| Model 1 + 2 | 890 | 0.8815 | 0.0206 | 135.44 |
| With temperature dropped | 877 | 0.8805 | 0.0001 | 118.78 |
| With exposure grade dropped | 893 | 0.8378 | 0.2462 | 110.49 |
| **Final model:**  **Anosmia + ageusia + smoking + symptomatic household members** | 889 | 0.8386 | 0.9655 | 111.71 |
